# A randomized sham-controlled trial on the effects of dual-tDCS “during” physical therapy on lower limb performance in sub-acute stroke and a comparison to the previous study using a “before” stimulation protocol

**DOI:** 10.1186/s13102-022-00463-9

**Published:** 2022-04-15

**Authors:** Wanalee Klomjai, Benchaporn Aneksan

**Affiliations:** 1 Neuro Electrical Stimulation Laboratory (NeuE), Faculty of Physical Therapy, Mahidol University, Salaya, Nakhon Pathom 73170 Thailand; 2 Faculty of Physical Therapy, Mahidol University, 999 Phuttamonthon 4 Road, Salaya, Nakhon Pathom, 73170 Thailand

**Keywords:** Dual-tDCS, Lower limb, Stroke, Physical therapy, Timing effect

## Abstract

**Background:**

Dual-transcranial direct current stimulation (tDCS) has been used to rebalance the cortical excitability of both hemispheres following unilateral-stroke. Our previous study showed a positive effect from a single-session of dual-tDCS applied before physical therapy (PT) on lower limb performance. However, it is still undetermined if other timings of brain stimulation (i.e., during motor practice) induce better effects. The objective of this study was to examine the effect of a single-session of dual-tDCS “during” PT on lower limb performance in sub-acute stroke and then compare the results with our previous data using a “before” stimulation paradigm.

**Method:**

For the current “during” protocol, 19 participants were participated in a randomized sham-controlled crossover trial. Dual-tDCS over the M1 of both cortices (2 mA) was applied during the first 20 min of PT. The Timed Up and Go and Five-Times-Sit-To-Stand tests were assessed at pre- and post-intervention and 1-week follow-up. Then, data from the current study were compared with those of the previous “before” study performed in a different group of 19 subjects. Both studies were compared by the difference of mean changes from the baseline.

**Results:**

Dual-tDCS “during” PT and the sham group did not significantly improve lower limb performance. By comparing with the previous data, performance in the “before” group was significantly greater than in the “during” and sham groups at post-intervention, while at follow-up the “before” group had better improvement than sham, but not greater than the “during” group.

**Conclusion:**

A single-session of dual-tDCS during PT induced no additional advantage on lower limb performance. The “before” group seemed to induce better acute effects; however, the benefits of the after-effects on motor learning for both stimulation protocols were probably not different.

*Trial registration* Current randomized controlled trials was prospectively registered at the clinicaltrials.gov, registration number: NCT04051671. The date of registration was 09/08/2019.

## Background

Transcranial direct current stimulation (tDCS) has been used as a priming modality in rehabilitation [1]. The anodal electrode can increase cortical excitability, while the cathodal electrode decreases it. The immediate effects of tDCS are to cause a shift of resting membrane potential and to induce changes in cortical excitability [2], while longer-lasting changes in corticospinal excitability are attributed to changes in synaptic efficacy [3]. Using tDCS for improving motor recovery after stroke is based on a theory of imbalance of two hemispheres (decrease of excitability of the affected hemisphere and an increase of excitability of the unaffected hemisphere), followed by an imbalance of interhemispheric inhibition (IHI) [4–6]. Dual-tDCS, anodal over the affected brain and cathodal over the unaffected side, has been used in stroke rehabilitation, aiming to restore cortical excitability and rebalance the IHI [7]. Dual-tDCS with motor rehabilitation treatments has been shown to benefit motor recovery [8–12]. Effects of a given tDCS protocol depend on several factors including intensity, duration, sessions, size of electrodes, montages, current density, and charge density [13, 14]. Other than these factors, tDCS delivered with a different timing relative to motor practice is an important regulatory component of priming and it has been claimed to induce differential effects on motor outcomes [15–20]. Stimulation protocols before and during motor practice were both widely used in tDCS studies [15]. However, the time at which the effect is the most induced is still a matter of conflict and the existing evidence in the lower limb motor recovery after stroke is relatively low [21, 22]. Motor learning plays an important role in the acquisition of skill required for motor function recovery after stroke. Animal studies have showed modulation of synaptic mechanism involved in learning process during tDCS application [23], and positive results on motor learning have been reported following tDCS applied during motor training in humans [24]. In the present study, we thus investigated the effect of a single-session of dual-tDCS combined with PT on lower limb muscle performance using a “during” stimulation paradigm. It was expected that long-term effects may occur, thus a follow-up of 1 week was performed.

Previously, our study showed that a single-session of dual-tDCS applied over the lower limb motor cortex before physical therapy (PT) acutely improved lower limb functions over sham in sub-acute stroke [8]. To promote lower limb motor recovery, it is still unknown whether tDCS applied during motor practice induce a greater effect. The comparison with the previous data from “before” stimulation protocol was then subsequently performed. This additional comparison would help to establish the optimal stimulation protocol for tDCS and to improve its effectiveness as an add-on intervention in stroke motor rehabilitation.

## Method

### Participants

Nineteen sub-acute stroke patients with hemiparesis (9 females, mean age 58.58 ± 2.64 years, age range 39–75 years) participated in the present study (see Table 1 for participant characteristics). They had first of all been diagnosed with cerebral infarction, confirmed by CT or MRI, with an onset of less than 6 months (average onset 3.3 ± 0.5 months). They were over 18 years old, able to understand the consent form and information sheet, and able to perform sit-to-stand and walk with or without gait aid for at least 3 m. They were screened for contraindications to tDCS and exclusion criteria, which were the presence of intracranial metal implants, cochlear implants, a cardiac pacemaker, history of seizures, no clear neurological antecedent history, or psychiatric disorder.

**Table 1:** Patient demographics of the present study and the previous study

	Present study “during”	Previous study “before”
Male/female	10/9	14/5
Age (years)	58.6 ± 2.6	57.2 ± 2.8
Onset (months)	3.3 ± 0.5	3.2 ± 0.4
Left/right hemiparesis	12/7	12/7
Lesion		
Cortical (MCA territory)	0	0
Subcortical (MCA territory)	18	14
Both (cortical and subcortical)	0	3
Brainstem	1	1
Unknown	0	1
Muscle strength evaluated by MMT		
III	0	7
III +	7	8
IV	7	4
IV +	5	0
Comorbidity diseases		
Hypertension	9	17
Diabetes mellitus	4	3
Dyslipidemia	5	6
Cardiovascular diseases		
Atrial fibrillation	0	1
Valve replacement	2	0
Cardiomegaly	1	1
No report	6	1
Receiving treatment during washout period (PT center/home exercises)	16/4	14/5

A description of the study was provided to all participants and written informed consent was obtained from all before the experiments. The study protocol was approved by the local ethics committee of Mahidol University. All methods were carried out in accordance with relevant guidelines and regulations. Our current study protocol was prospectively registered at the clinicaltrials.gov (ID number: NCT04051671, date of first registration 09/08/2019). Data collection was performed at the Physical Therapy Center, Mahidol University.

### Experimental protocol

**Fig. 1:**
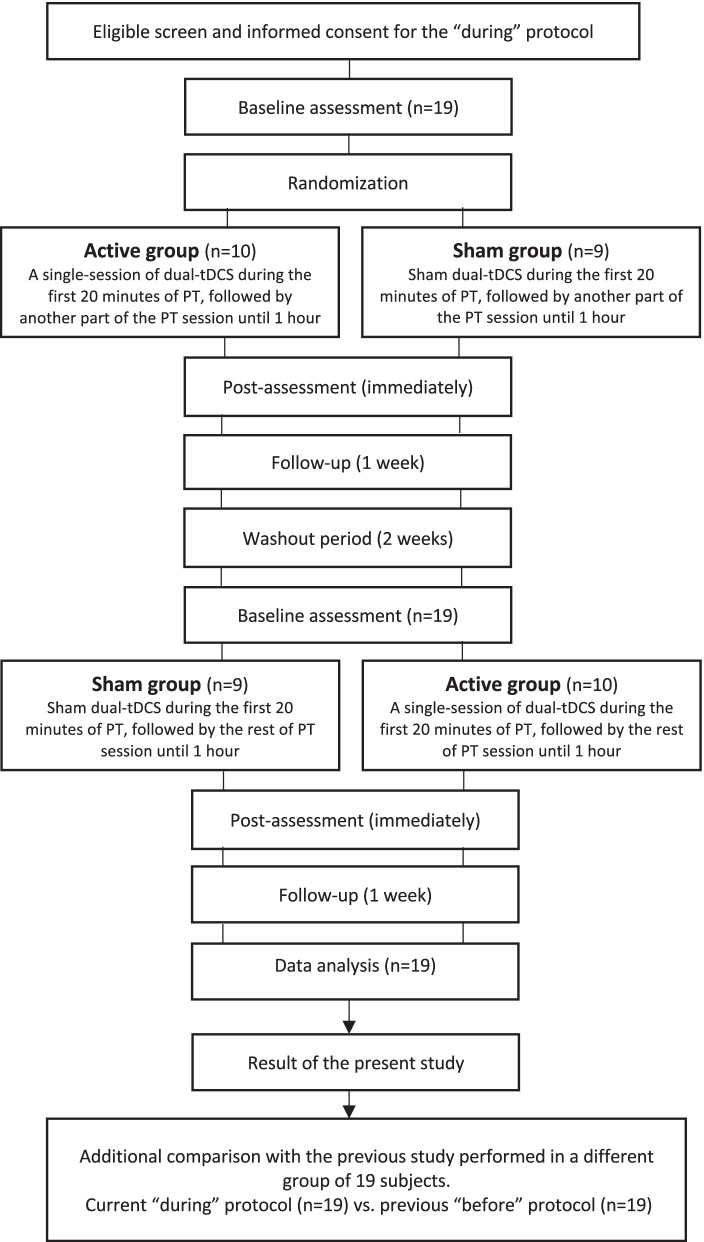
Flowchart of study procedure of the present study

The present study (“during” protocol) was conducted as a randomized sham-controlled crossover trial. This was the same experimental protocol as our trial for the “before” stimulation paradigm [8]. Each participant completed two sessions of experiments (active/sham tDCS) in random order. Randomization occurred through an application number sequence by an independent researcher who was not involved in the tDCS administration or outcome assessments. Participant, rater, and physical therapist were blind to the treatment allocations. The wash-out period was at least 2 weeks between treatment periods since a previous study showed that an after-effect of a single 20 min session of tDCS combined with rehabilitation treatments disappeared at 11-day post-intervention [25]. For the “during” stimulation paradigm, participants received a single-session of dual-tDCS (active/sham) during the first 20 min of PT. After tDCS, the PT session continued until 1 h. In our previous study, participants received dual-tDCS for 20 min and the PT session was started immediately after tDCS for an hour. None of the participants participated in both current and previous studies (see Fig. 1. Flowchart of study procedure).

To assess the lower limb performance, we used the same outcome measures as our previous study [8]. The Timed Up and Go test (TUG) and Five-Times-Sit-To-Stand test (FTSTS) were performed at PRE-, POST-intervention and follow-up (F/U) at 1 week by a rater who was blind to the intervention. Participants were asked to perform two trials for each test and the best trial was chosen. Both tests have been reported as reliable [26, 27]. The TUG is commonly used in clinical practice to assess the lower limb muscle strength and gait performance [26]. To perform the TUG, participants were asked to sit on a chair and place their back against the chair. Timing started after participants left the chair, whereupon they walked for 3 m, turned, walked back and sat down. Timing ended when the back was against the chair again. Times were recorded in seconds. A high score on the TUG indicates a poor performance and had a significant higher risk of falling after stroke [28, 29] for example, stroke patients with TUG time of ≥ 15 s were at higher risk of falling [28]. The minimal detectable changes (MDC) of the TUG was reported as 3.2 s in stroke [30]. The FTSTS is commonly used as a measure of lower limb strength, balance, and exercise capacity [31]. Participants were asked to perform sit-to-stand 5 times as quickly as possible from a sitting position to standing up with their legs fully extended. Timing began after they left the chair and ended when they seated after the fifth sit-to-stand. A high score on the FTSTS indicates a poor performance. Cut-off scores of 12 s was found to discriminate between healthy, elderly and stroke people [27]. The MDC of the FTSTS was reported in stroke as 1.14 s [27, 32].

### Intervention

#### Dual-tDCS

Skin preparation was required before applying the electrodes. The tDCS (Ybrain, MINDD STIM, Korea) delivered a direct current through two rectangular saline-soaked sponge pad electrodes of 35 cm^2^ surface area. Current intensity was fixed at 2 mA, thus the current density was 0.057 mA/cm^2^. Anodal tDCS was applied over the motor cortex (M1) of the affected hemisphere, while cathodal tDCS was placed over the M1 of the unaffected hemisphere with the medial border of each electrode placed 5 mm lateral from the vertex using a 10–20 EEG system [8]. The electrodes were firmly attached using a headband and head cap. An auto alarm system for tDCS is available to indicate if electrodes detach during physical therapy treatment or high impedance occurs, whereupon the current will then be immediately stopped. The current flowed continuously for 20 min during the real condition and for the first 30 s during the sham condition. The sham mode is an auto mode and can be chosen in the configuration system, the current will be automatically stopped after the first 30 s without removing the electrodes until 20 min. The tDCS application was performed by a researcher who was blind to the outcome assessment. Any adverse effects were also recorded during stimulation.

#### Conventional PT

Participants received PT from a physical therapist who was blind to the tDCS intervention. The physical therapist was not the same person as in our previous study; however, the physical therapists in both studies have 5–10 years of experience with stroke rehabilitation and they were trained to provide treatment with this program. The PT session was administered for an hour to improve the strength of the affected lower limbs, balance, and gait as follows: (1) active stretching for about 10 min (hold for 10 s/time, 10 times/set) for hip flexors, hip extensor, hip internal rotator, knee extensor, and ankle plantar flexor; (2) strengthening exercises of hip flexor, hip extensor, knee extensor, and ankle muscles (15 times/set, 2 sets/muscle); (3) step training (step forward–backward, step sideways, step up-down) 10 times each, and (4) gait training for about 10 min. This moderate-intensity exercise program (evaluated by the rating of perceived exertion [33] and order of exercises were the same for all participants and were similar to the previous study [8].

### Sample size calculation

To evaluate the outcome measures under interventions between groups (active vs. sham) for the present study, sample size for comparing the two means was calculated based on a previous work on the effect of tDCS with physical therapy on lower limb performance in acute-subacute stroke [34], a significance level of α = 0.05 and 80% power, resulting in 19 participants per group to which 20% was added to compensate for possible dropouts. Thus, the sample was composed of 19 in the active tDCS group and 19 in the sham group for the present study (“during” protocol).

### Data analysis

- To compare “during” versus sham in the present study*,* two-way repeated ANOVA was used.
- To compare with “before” stimulation*,* the difference of mean changes of outcome measures from individual baselines were analyzed by using the equations as follows: (1) PRE minus POST, and (2) PRE minus 1-week F/U. We used data only from the active tDCS group of our previous cross-over sham-controlled study [8] as there were no significant differences between sham groups of the two studies. The Mann–Whitney U test or ANOVA was used to compare data among the groups at the baseline. Depending on the normality of the data distribution, statistical differences were estimated by ANOVA followed by Tukey’s post hoc analysis or Kruskal–Wallis one-way ANOVA on ranks followed by Tukey’s test or Dunn’s method. Significance was set at *P* < 0.05. Means are reported ± SEM. Statistical analyses were performed using Sigma Plot.

## Results

Participants were asked about their feelings during tDCS. For the active group, 79% of participants reported cutaneous sensations (i.e., itching, tingling, burning) during stimulation, and some participants felt a decline/disappearance of sensation at the late period of stimulation. For the sham group, 32% of participants reported cutaneous sensations during the early period of stimulation. None of the participants reported any adverse effects after tDCS removal.

### “During” stimulation v Sham

We performed two-way repeated ANOVA with the stimulation type (dual and sham) as a between-subject factor and the time (PRE, POST, F/U) as a within-subject factor.

**FTSTS**: For the active group, the FTSTS score at PRE was 16.95 ± 1.37 s, POST was 16.60 ± 1.23 s, and F/U was 15.18 ± 0.98 s. For the sham group, the FTSTS score at PRE was 14.87 ± 1.17 s, POST was 14.80 ± 1.13 s, and F/U was 14.71 ± 1.28 s (see Table 2). There was no significant difference at the baseline (*P* = 0.226). Two-way repeated measures ANOVA showed insignificance for the effects of Time (F_(1,18)_ = 2.211, *P* = 0.124), the effect of Stimulation (F_(1,18)_ = 2.708, *P* = 0.117), and interaction between Time and Stimulation (F_(1,18)_ = 2.027, *P* = 0.147).

**Table 2:** Raw means data of FTSTS and TUG in seconds expressed as mean ± SEM evaluated at PRE, POST and F/U at 1 week in each group (bolditalic). Differences from PRE (PRE–POST and PRE–F/U) and *P* value (italic)

Outcome measures	Group	Mean ± SEM (s)			Diffrences from PRE		*P* value			
							POST		F/U	
		PRE	POST	F/U	POST	F/U	overall	Post hoc	overall	Post hoc
FTSTS	Sham	***14.87* ± *1.17***	***14.80* ± *1.13***	***14.71* ± *1.28***	*0.06* ± *0.38*	*0.15* ± *0.66*		*B* > *S (P* < *0.05)*		
	During	***16.95* ± *1.37***	***16.60* ± *1.23***	***15.18* ± *0.98***	*0.35* ± *0.72*	*1.77* ± *0.76*	*P* ≤ *0.001*^*a*^	*B* > *D (P* < *0.05)*	*P* = *0.015*^*b*^	*B* > *S (P* = *0.011)*
	Before	***16.74* ± *1.19***	***13.68* ± *0.87***	***14.49* ± *0.99***	*3.06* ± *0.64*	*2.25* ± *1.02*				
TUG	Sham	***21.66* ± *3.33***	***21.21* ± *3.16***	***21.06* ± *3.01***	*0.45* ± *0.93*	*0.60* ± *1.24*		*B* > *S (P* < *0.05)*		
	During	***22.38* ± *2.67***	***22.15* ± *2.70***	***19.77* ± *2.58***	*0.23* ± *1.10*	*2.61* ± *1.02*	*P* = *0.003*^*a*^	*B* > *D (P* < *0.05)*	*P* = *0.024*^*a*^	*B* > *S (P* < *0.05)*
	Before	***21.41* ± *2.85***	***17.75* ± *2.01***	***18.53* ± *2.83***	*3.66* ± *1.18*	*2.87* ± *2.10*				

^a^Testing by Kruskal–Wallis one-way ANOVA by ranks

^b^Testing by One way ANOVA; S = Sham; D = During; B = Before; PRE: pre-intervention; POST: post-intervention; F/U: follow-up. Significant level at *P* < 0.005

**TUG**: For the active group, the TUG score at PRE was 22.38 ± 2.67 s, POST was 22.15 ± 2.70 s, and F/U was 19.00 ± 2.58 s. For the sham group, the score at PRE was 21.66 ± 3.33 s, POST was 21.21 ± 3.16 s, and F/U was 21.06 ± 3.01 s (see Table 2). There was no significant difference at the baseline (*P* = 0.602). Two-way repeated measures ANOVA showed insignificance for the effects of Time (F_(1,18)_ = 2.480, *P* = 0.098); the effect of Stimulation (F_(1,18)_ = 0.0134, *P* = 0.909), and interaction between Time and Stimulation (F_(1,18)_ = 1.473, *P* = 0.243).

### Comparing with previous data, “before” stimulation

The previous data were collected in 19 sub-acute stroke patients at PRE and POST and in only 10 participants for 1-week F/U. Both studies had similar age, time after stroke, lesion, and paretic side (Table 1). Baseline muscle strength evaluated by MMT showed differences between the two studies (Mann–Whitney U test, *P* ≤ 0.001). The baseline performances of FTSTS and TUG were not significantly different between “before”, “during” and “sham” (Kruskal–Wallis one-way ANOVA by ranks, *P* > 0.05).

**FTSTS**: Data from the “before” stimulation at PRE was 16.74 ± 1.19 s, POST was 13.68 ± 0.87 s, and F/U was 14.49 ± 0.99 s.

**Fig. 2:**
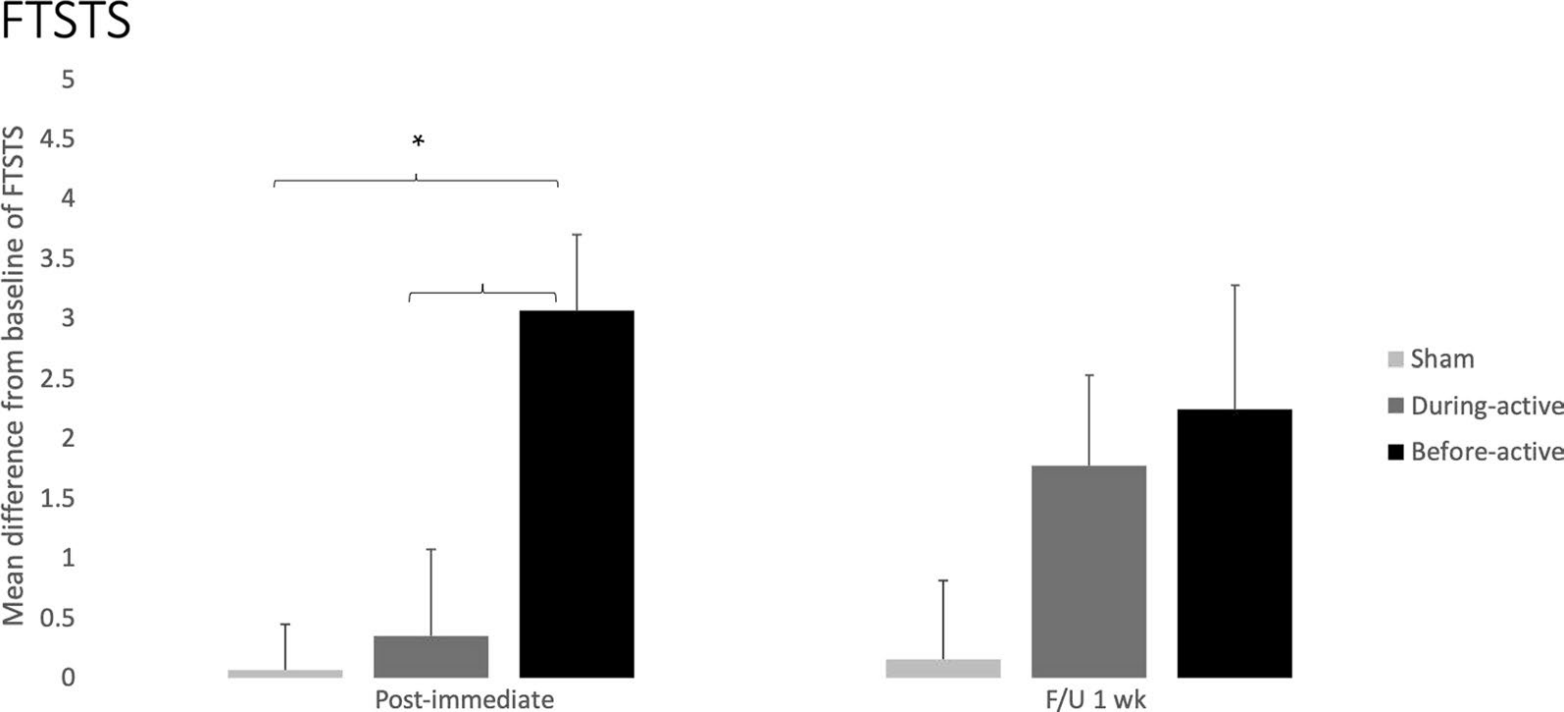
The column graph represents mean differences from baseline (PRE–POST) and (PRE–F/U) of FTSTS for each group. Vertical bars represent the standard error of the mean. Asterisks represent significant differences of *P* < 0.05 (*)

**PRE–POST**: The change score for PRE–POST of the group “before” was 3.06 ± 0.64 while for the “during” and sham groups the scores were 0.35 ± 0.72 and 0.06 ± 0.38, respectively (Fig. 2), for individual data, see Table 3A. At post-intervention, Kruskal–Wallis one-way ANOVA by ranks revealed a significant difference among the groups (H = 16.114, *P* ≤ 0.001) and Tukey's test for comparison between groups showed significant differences between “before” versus sham (q = 5.169, *P* < 0.05), and between “before” versus “during” (q = 4.616, *P* < 0.05).

**Table 3:** Individual data represents differences from baseline (PRE–POST) and (PRE–F/U) for FTSTS (A) and for TUG (B). (A) The FTSTS data in bold represent if the value is equal or more than 1.14 s as it has been reported as a minimal detectable change in stroke (27,32), (B) The TUG data in bold represent if the value is equal or more than 3.2 s as it has been reported as a minimal detectable change in stroke (30)

Subject	Sham		During		Subject	Before	
	PRE–POST	PRE–F/U	PRE–POST	PRE–F/U		PRE–POST	PRE–F/U
*(A) FTSTS*							
D01	0.22	0.33	**1.41**	− 5.81	B01	**1.91**	**2.95**
D02	**3.03**	**2.48**	1.08	**4.33**	B02	0.6	
D03	**2.91**	0.59	− 0.09	**2.42**	B03	− 0.06	
D04	0.39	0.33	− 0.5	− 1.43	B04	**5.73**	**1.71**
D05	**3.54**	**4.69**	**6.76**	**7.76**	B05	**2.34**	− 0.38
D06	− 1.92	− 4.56	**1.7**	**1.41**	B06	**3.3**	**3.14**
D07	− 1.58	− 1.22	− 2.62	**3.18**	B07	**4.76**	
D08	− 1.62	**3.18**	**2.04**	**5.56**	B08	1.04	
D09	− 1.49	− 5.87	**7.98**	**7.07**	B09	**1.51**	
D10	− 0.11	**1.74**	− 1.37	**2.24**	B10	**10.11**	
D11	− 1.63	**1.52**	− 0.91	0.67	B11	**1.56**	**8.1**
D12	− 0.73	0.14	− 1.16	− 1.1	B12	**8.35**	**10.12**
D13	− 1.19	− 5.36	− 6.51	0.55	B13	0.09	1.03
D14	− 0.58	− 1.08	**1.85**	**2.18**	B14	**3.47**	
D15	**1.42**	**2.57**	− 1.28	**4.74**	B15	**1.51**	
D16	0.57	**3.32**	0.06	**1.14**	B16	**2.68**	
D17	− 0.04	0.76	− 1.28	− 0.27	B17	**2.55**	**3.15**
D18	− 0.71	− 0.56	0.25	**1.15**	B18	1.05	**2.79**
D19	0.75	− 0.1	− 0.76	− 2.09	B19	**5.77**	**5.84**
*(B) TUG*							
D01	0.62	− 0.22	0.86	0.23	B01	**3.62**	**5.48**
D02	1.40	− 2.00	− 0.27	1.98	B02	0.75	
D03	**4.22**	**6.68**	0.07	2.89	B03	0.87	
D04	− 8.62	− 2.41	− 8.19	− 4.38	B04	2.38	− 2.16
D05	− 0.57	− 4.31	**11.57**	2.45	B05	− 0.07	**5.43**
D06	− 0.87	1.10	0.54	0.7	B06	**8.91**	**8.26**
D07	− 2.40	− 5.32	− 2.87	− 1.17	B07	2.22	
D08	1.02	− 2.81	**9.52**	**11.04**	B08	1.16	
D09	**12.93**	**19.78**	− 0.9	− 0.18	B09	0.62	
D10	0.11	− 0.78	− 0.26	**14.87**	B10	1.47	
D11	0.44	2.14	0.58	2.43	B11	**21.26**	**19.51**
D12	− 1.16	− 0.87	0.69	2.28	B12	**10.83**	**12.49**
D13	2.07	0.01	2.64	**3.33**	B13	0.82	− 1.63
D14	0.91	2.32	**3.88**	**6.39**	B14	1.71	
D15	− 2.74	0.50	− 8.65	2.88	B15	0.6	
D16	2.08	1.56	− 1.78	− 0.45	B16	**4.75**	
D17	1.34	− 3.48	− 3.18	**5.22**	B17	1.95	1.92
D18	0.40	0.49	− 0.46	− 1.29	B18	1.47	1.43
D19	− 2.64	− 0.94	0.61	0.33	B19	**4.2**	**3.88**

**PRE–F/U**: The change score PRE–F/U of the group “before” was 2.25 ± 1.02, while for the “during” and sham groups they were 1.77 ± 0.76 and 0.15 ± 0.66, respectively (Fig. 2), for individual data, see Table 3A. At follow-up, One way ANOVA revealed a significant difference between groups (F_(2,45)_ = 4.625, *P* = 0.015). Tukey’s test showed a significant difference between “before” versus sham (q = 4.281, *P* = 0.011).

**TUG**: Data from the “before” stimulation at PRE was 21.41 ± 2.85 s, POST was 17.75 ± 2.01 s, and F/U was 18.53 ± 2.83 s.

**Fig. 3:**
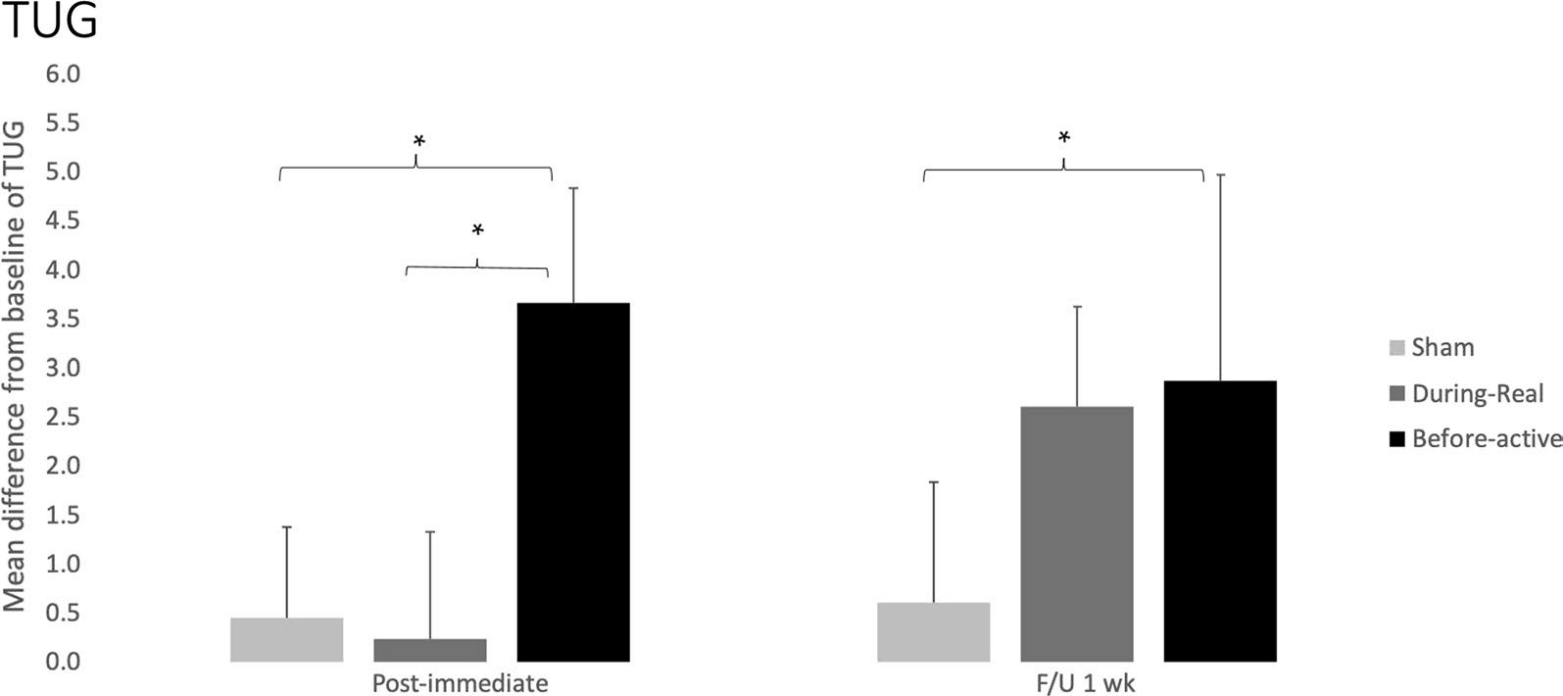
The column graph represents mean differences from baseline (PRE–POST) and (PRE–F/U) of TUG for each group. Vertical bars represent the standard error of the mean. ‬‬‬Asterisks represent significant differences of *P* < 0.05 (*)

**PRE–POST**: The change score for PRE–POST of the group “before” was 3.66 ± 1.18 while for the “during” and sham groups the scores were 0.23 ± 1.10 and 0.45 ± 0.93, respectively (Fig. 3), for individual data, see Table 3B. At post-intervention, Kruskal–Wallis one-way ANOVA by ranks revealed a significant difference among the groups (H = 11.578, *P* = 0.003) and Tukey's test showed significant differences between “before” versus sham (q = 3.787, *P* < 0.05), and between “before” versus “during” (q = 4.464, *P* < 0.05).

**PRE–F/U**: The change score PRE–F/U of the group “before” was 2.87 ± 2.10 while for the “during” and sham groups the scores were 2.60 ± 1.01 and 0.60 ± 1.24 respectively (Fig. 3), for individual data, see Table 3A. At follow-up, Kruskal–Wallis one-way ANOVA by ranks revealed a significant difference among the groups (H = 7.455, *P* = 0.024) and Dunn's test showed significant differences between “before” and sham (q = 2.532, *P* < 0.05).

## Discussion

The present results showed that dual-tDCS “during” PT did not significantly improve lower limb performance at post-intervention and at 1-week F/U; no significant changes were observed in either “during” active or sham groups and no significant difference was found between the groups. The data were then compared to those from the “before” tDCS study [8]. At post-intervention, “before” tDCS significantly induced greater changes compared to “during” and sham. While at 1-week F/U, the “during” group continued to improve from post to follow-up on both outcome measures, whereas the “before” study showed a small decline. In the end, it appears that PRE–F/U data of both outcomes were not significantly different between the “before” and “during” groups, and the “before” group was only greater than the sham.

Dual-tDCS reportedly rebalances IHI [35], induces greater lower limb motor recovery than unilateral-tDCS [14, 36], and produces a more prominent effect on lower limb function in the subacute phase of stroke [21]. However, we found non-significant lower limb motor improvement following dual-tDCS “during” PT in subacute stroke in the present study. This could be due to several reasons. It should be noted that for the “during” stimulation paradigm, tDCS was applied concurrently only with the first 20 min of the PT session and was followed by another part of the PT session until 1 h, not all time concurrent with tDCS. Studies that reported positive effects after the “during” tDCS stimulation paradigm used a real concurrent-tDCS, i.e., motor practice for 10–30 min during tDCS application [17–19]. However, we investigated tDCS effect with PT in the real clinical setting since a PT session is recommended for 45 min-1 h for stroke rehabilitation [37]. Second, for almost the first 10 min of the PT session, the participants had to perform stretching exercises that were concurrent with tDCS. This would probably interfere with the tDCS effect since it was reported that non-exhaustive active and passive movements decreased MEPs amplitude during tDCS [38]. In particular, MEPs amplitudes were reduced during muscle lengthening [39–41] with suppression being more evident at higher stimulation intensities [40]. It is speculated that this reduction of cortical excitability is caused by an activation of Ia afferent input during muscle lengthening [39–41]. Therefore, an appropriate PT program for the “during” tDCS stimulation paradigm needs to be carefully considered in further studies. Third, it can be argued that any change would be seen immediately following stimulation period but it was missed by testing at the end of PT session. However, previous studies have shown that the effects of a single session of anodal tDCS were observed immediately or within 10 min following the end of stimulation which continues through later time points [17, 42, 43], and that could be found at post 24 h when combined with motor training [17]. Next, a considerable inter-individual variation was also observed following tDCS over the leg motor area [44, 45]. This may contribute to our non-significant difference results at a between-group level. It appears that this considerable inter-individual variability is due to a variety of individual factors such as gender [46], hormonal influences [47–49], genetic [50], and anatomy [51]. Based on patients’ characteristic, it appeared that most patients in the present study had relatively high performance regarding lower limb muscle strength (12 from 19 participants had grade IV–IV +), see Table 1. It has been shown that tDCS-induced motor improvement was more pronounced in stroke people with more impairment in the upper limbs [52, 53] and lower limbs [54]. This may cause a non-significant improvement over sham following “during” stimulation paradigm. In addition, it can be argued that patients with more comorbidities were associated with worse functional outcome since comorbidity was a good predictor of the effectiveness of rehabilitation treatment [55]. Previous studies showed that myocardial infarction had a considerable impact on the motor outcomes of stroke patients [56, 57], while an impact from atrial fibrillation was indirect [58]. Diabetes mellitus and hypertension were not predictors of rehabilitation outcome for stroke [57]. In our study, most of comorbidity diseases of participants were hypertension and there was no report of myocardial infarction (see Table 1).

Our comparison of “before” versus “during” with mean difference changes suggested that the order of tDCS application could have differential acute effects, but not for post-effects. The immediate effects of tDCS are due to changes in membrane potential, while long-lasting changes in cortical excitability are attributed to changes in synaptic efficacy that depend on glutamatergic mechanisms [3]. The two approaches (before or during motor practice) have different proposed mechanisms. More relevant to our “before” stimulation paradigm is motor priming during the resting state of corticospinal neurons. Recently, it was reported that priming the M1 with anodal tDCS before a single session of strengthening exercise reduced corticospinal inhibition (GABA-mediated inhibitory projections) that results in enhanced synaptic plasticity, without inducing changes in corticospinal excitability [59]. Meanwhile, gating mechanisms (weakening the excitability of intracortical inhibitory circuits) have been proposed as a principle of motor priming when the stimulation is applied concurrently with motor exercises or immediately before exercises [60]. This is more relevant to our “during” stimulation paradigm. At present, there are conflicting results regarding the timing of brain stimulation relative to the learning period. Giacobbe et al. demonstrated in chronic stroke that upper limb movement smoothness immediately improved by about 15% with anodal-tDCS (2 mA, 20 min) delivered before motor practice, while anodal-tDCS delivered during motor practice did not offer any benefit and reduced speed when delivered after practice [61]. Cabral et al. reported in healthy subjects that anodal tDCS (1 mA, 13 min) before finger motor tasks increased MEP amplitudes, while MEPs remained unchanged when tDCS was administered during or immediately after the motor practice [16]. They also claimed that activation of regulatory homeostatic mechanisms was probably a cause of the absence of tDCS-induced MEPs. While some studies reported the benefit of concurrent tDCS over the before stimulation paradigm, Stagg et al. showed that tDCS (1 mA, 10 min) during motor tasks led to the modulation of behaviour in a polarity specific manner, while tDCS before performance led to slower learning after both anodal and cathodal tDCS [18]. These findings matched those of Jin et al. who showed in chronic stroke that 5-session dual-tDCS (1 mA, 30 min) with mirror therapy improved upper limb function in the during-tDCS group compared to the before-tDCS and sham-tDCS groups at post-intervention, however no significant differences were found at F/U 2-weeks [19]. Sriraman et al. investigated in healthy subjects and found that dual-tDCS (2 mA, 25 min) application during practice of a skilled ankle motor task increased motor performance to a greater extent than tDCS applied before the motor practice, however at post-24 h, both dual-tDCS groups showed the same enhanced motor learning [17]. Taking these together, it seems that both stimulation paradigms induce similar post-effects as observed in the present study, while the acute effect is still under debate. A systematic review and meta-analysis reported that stimulation protocols before and during motor practice were both used in tDCS studies published from 2005 to 2015 [15]. This review compared timing stimulation effects in 21 tDCS studies in the upper limbs. The results showed that the effect size (ES) for stimulation before motor practice was 0.70, which was slightly higher than that of stimulation during motor practice (ES = 0.53). However, these results indicated beneficial effects on long-term motor learning for both stimulation protocols. In addition, considering the minimal detectable changes (MDC), the numbers of participants who achieved the MDC in the present study were not obviously different between the “before” and “during”. For FTSTS, the number of participants who achieved the MDC were 8 in the sham group, 13 in the “during” group, and 15 in the “before” group (see data in Table 3A). For TUG, the number of participants who achieved the MDC were 2 in the sham group, 6 in the “during” group, and 7 in the “before” group (see data in Table 3B).

Given the present results, it is still unknown whether the dual-tDCS “before” PT could be considered clinically beneficial in terms of effects compared to the “during” group. From the clinical advantage aspect, dual-tDCS during PT treatment would be more time-saving, however, it needs greater care to avoid electrode dislocation during exercises of the lower limb, unless using a fit cap with an alarm system to signal electrode dislocation as used in the present study. Moreover, as cutaneous sensations (i.e., itching, tingling, burning) were reported during stimulation, it is interesting to further explore whether those sensations disturb the participant’s concentration during motor practice. However, we kept giving them commands to focus on exercises during PT session.

### Study limitations

A major limitation of this study is the study design that was used to compare the effect of “before” versus “during”. A stronger comparison, i.e., randomized sham-controlled crossover design with a larger sample size, should be considered in further studies. Second, the participants in both studies were assessed at about 3 months after stroke onset (see Table 1). The time effect in natural recovery within/over 3 months after stroke onset may be an affecting factor. However, participants in the current study were randomly assigned into both active and sham groups in the order “active-sham” (10 participants) and “sham-active” (9 participants) the same as in the “before” study. This randomization was required to avoid this impact. Third, some data were lacking at F/U in the “before” group. Forth, most participants from both studies were recruited from our Physical Therapy Center, and they were allowed to receive regular conventional PT rehabilitation (approximately 3 days per week) or to perform exercises at home during the washout period. These factors may affect the result. However, the number of participants who received those treatments was not different between both studies (see Table 1) and the sham-controlled crossover design used in both studies could help to reduce this impact. In addition, participants were not allowed to receive alternative treatments such as acupuncture, TMS, etc. during their participation. Lastly, there was a lack of neurophysiological examination (i.e. cortical excitability assessment) regarding the effects of tDCS. However, some studies have reported changes of motor behavior induced by tDCS with non-significant changes in corticomotor excitability [43, 62]. Lastly, it has been mentioned that flunarizine, a T-type calcium-channel blockers (which is mainly used to treat vertigo [63], migraine [64], and epilepsy [65]) diminished the effects of anodal tDCS in humans brain [66]. In the present study, antihypertensive drugs (such as amlodipine, manidipine, and nicardipine) which are L-type of calcium channels [67] were used in participants who had hypertension. These antihypertensive drugs mainly affect vascular smooth muscles and cardiac muscles which active on different types of calcium channels compared to flunarizine.

## Conclusion

A single-session of dual-tDCS applied during PT conferred no additional advantage on lower limb performance compared to the sham. By comparing with our previous study, it seems likely that applying tDCS “before” could be of greater benefit in terms of acute effects, but the stimulation paradigms showed no difference in terms of after-effects. Nevertheless, the comparison of “before” versus “during” has some limitations (i.e., study design) that should be addressed in further studies. For practical application, during-tDCS would be more time-saving. However, if during-tDCS is chosen in further studies, possible displacement of electrodes during motor practice and adverse effects occurring during tDCS that may disturb the motor practice are issues to be aware of. Moreover, the type of exercise used during tDCS would need to be carefully considered.

## Data Availability

All data generated or analysed during this study are included in this published article.

## Abbreviations

tDCS: Transcranial direct current stimulation
IHI: Interhemispheric inhibition
PT: Physical therapy
M1: Primary motor cortex
TUG: Timed Up and Go test
FTSTS: Five-Times-Sit-To-Stand test
F/U: Follow-up
